# Optimization of roadway layout in ultra-close coal seams: A case study

**DOI:** 10.1371/journal.pone.0207447

**Published:** 2018-11-21

**Authors:** Gang Wu, Xinqiu Fang, Hualin Bai, Minfu Liang, Xiukun Hu

**Affiliations:** Key Laboratory of Deep Coal Resource Mining, Ministry of Education of China, School of Mines, China University of Mining & Technology, Xuzhou, China; Central South University, CHINA

## Abstract

Most coal mines in China are currently mining close coal seams. Roadways in close coal seams, especially ultra-close coal seams, confronted difficulties in maintaining, including large deformation of the roadway, roof caving, rib spalling and floor heaving. This is mainly caused by the complicated stress and geological conditions, shattered roof, improper layout and support. To explore the issues mentioned above, the theoretical analysis was used to build a mechanical model and study the stress distribution under coal pillars, and FLAC3D modelling was adopted to build numerical models with different staggered distances. The optimal roadway layout was brought forward combining the result of numerical simulation and coal recovery rate. The field practice was carried out in the tailgate of panel 25301 to investigate the effect of the layout scheme. The results of field monitoring show that the roadway’s stability is well maintained in the mining process.

## 0 Introduction

China is the largest coal producer and consumer in the world, and according to the BP Statistical Review of World Energy 2015 program, 47.4% of global coal production (3,874 out of 8,165 million tons) is from China in year 2014. More than 90% of Chinese coal output is produced by underground coal mines [1], many of which has multiple coal seams [2]. Over the past decades, large-scale coal mining activities have led to the depletion of shallow coal resources. Therefore, coal mining gradually turned to deep and multi-seam mining [3, 4]. Multi-seam mining encounters complex geological and stress conditions caused by adjacent coal seam mining, and is more prone to more mine pressure phenomena., such as coal wall spalling, floor heaving, and roadway deformation, etc[5, 6]. Thus the mining of close seams draw the attention of scholars and field engineers in past years [7–11].

In multi-seam mining, the distance between upper and lower seams is the dominant factor in the mutual influence of coal seams [12]. From the mining operation point of view, the closer the distance between two seams, the more serious the impact of the upper seam on the lower seam [13]. This impact, which include not only the effect on surrounding rock control in stope, but also the impact on the lower seam mining method and roadway layout as well, is especially more severe when the coal seams is ultra-close. In longwall mining, the roadways of the lower seam are usually located under the goaf area of upper seam to reduce the pressure around the adjacent rock mass. However, the roof of the lower seam roadways can be damaged or even shattered by upper panel mining process, hence, gas and water can flow through the fractured roof into the lower stope, causing potential hazards.

For the purpose of maintaining working face stability and isolating gas stored in gob area, coal pillars are set up between working faces, usually with a width of tens of meters, which cause stress concentration in lower roadways and working faces, and the roadway stability concerns conveyance, ventilation and pedestrians, etc. [14]. In close coal seams mining, the layout, i.e., relational position to upper seam roadway with staggered distance, of lower seam roadway and corresponding support system is the decisive factor for roadway stability, maintenance difficulty and the safety of personnel involved during the process of roadway extraction and coal mining of the lower seam. Besides, an optimal layout of lower seam roadway combined with proper support scheme can save the cost of repeated repair, mitigate greatly the potential hazards of roof fall and rib spalling, and improve the safety of personnel involved as well. However, when the position of lower seam roadway is inappropriately designed or the roadway lack sufficient support strength, the surrounding rock may deform severely under the influence of mining pressure and increase the possibility of burst-instability, especially when the roadway is right beneath the coal pillar left in upper seam. Besides all that, under the influence of static and mining-induced dynamic stress, the isolated coal pillars are potential of rock burst which will cause instability in working faces and roadways of lower seams [11, 15].

This paper reports a case study on roadway layout at an underground coal mine in Shanxi Province, China, where maintaining roadway stability has been a challenging task due to roof damage by upper mining operations and roof stress concentration caused by upper isolated coal pillars. Mine-site stress calculation and numerical simulations are adopted to systematically investigate the optimal layout of the roadways in lower seam, and then field practice and monitoring of the roadway deformation are conducted to validate the effect of the layout scheme.

## 1 Site description for Shaqu No.1 coal mine

**Fig 1:**
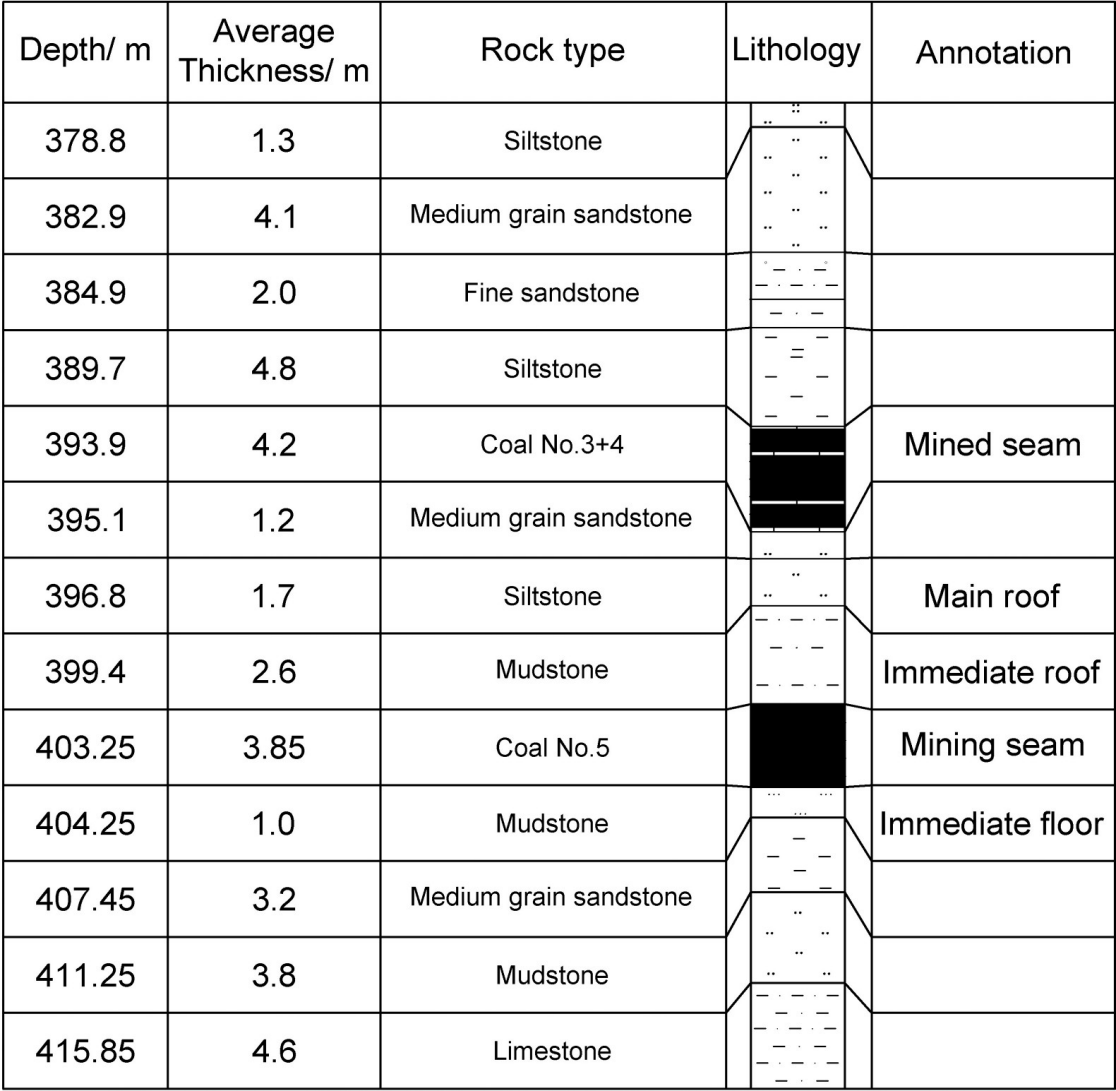
General stratigraphic log of panel 25301.

Shaqu No.1 coal mine is located in Lvliang city (N37°24′49.58″, E110°51′49.28″), Shanxi Province, China, with the production capacity of 5Mt/a and the coal mine mainly produces high-quality coking coal. It has 8 workable coal seams in total, of which coal seams No.2, 3, 4, and 5 have relatively small separations. Coal seam No.3 and No.4 have a spacing of dozens of centimeters, hereby are referred as seam No.3+4, and mined as a single seam as well. Coal seam No.3+4 and No.5 have high gas content, and are pre-drained before the mining activity. Coal seam No.3+4 has an average thickness of 4.62m, a dip angle of approximately 4° and buried depth of around 400m, while the thickness and angle of seam No.5 are 3.85m and roughly 5° in the area of panel 25301, respectively. The coal mine is currently in the first level of +400m, and working on seam No.3+4 and seam No.5 seams, whose average interlayer distance is merely 5.5m. The stratigraphic sequence of the test areas is shown in Fig 1.

**Fig 2:**
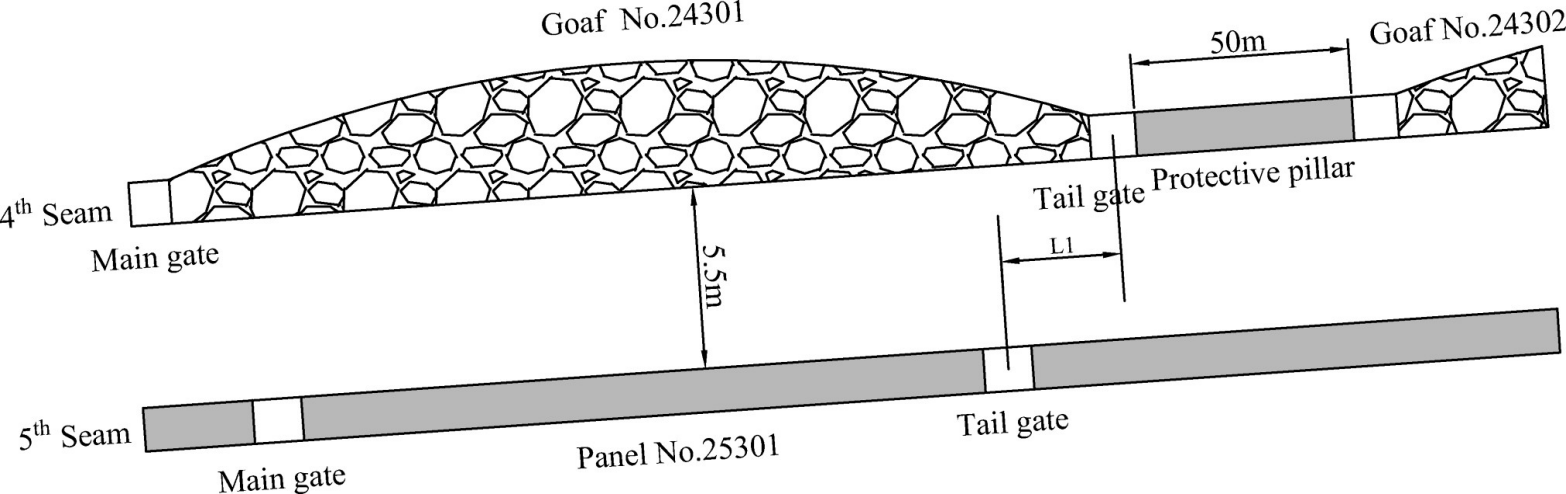
Schematic representation of goaf 24301 and 24302, and roadway layout of panel 25301.

Panel 24301 and 24302 are mined before the mining of coal seam 5th, and the isolated protective pillar between these two panels is 50m wide. Panel 25301 is designed to be laid out under goaf No.24301 and the next goaf (No. 24302). For clarity, the whole layout scheme is shown in Fig 2.

## 2 Definition of ultra-close coal seams

Currently, the definition of close distance seams is not clear and lacks quantitative indicators. According to the document safety rules in Coal Mines issued by China’s Administration of Coal Mine Safety (March, 2011), close seams are defined as “coal seams with small separations that exert great influence over each other in the mining process”[16]. Zhang[13] and Yuan et al[17] defined ultra-close multiple seams as those with interlayer distance less than or equal to the floor damage depth caused by the mining process of the upper coal seam. And this definition can be expressed in the following equations:

$$h_{j}\leq f_{s}∙h_{\sigma }$$ (1)

Where, *h*_*j*_ is the distance between coal seams; *f*_*s*_ is the safety factor. In this paper, the abovementioned definition was adopted.

Initial stress in the vicinity of coal seams is in a state of equilibrium, while excavation of coal seam causes stress redistribution in floor strata. Stress in some area of the floor is released, and concentrated in other areas. The stress concentration in the floor strata causes original cracks in rock to propagate and conjugate, resulting in floor strata damage. The maximum depth of floor strata damage (*h*_*σ*_) is determined by many factors, including depth, thickness, dip angle, of coal seam, and Protodyakonov strength of floor strata[18]. National Coal Industrial Bureau [19] gives an empirical formula for the calculation of floor strata damage depth:

$$h_{\sigma }=0.0085H+0.1665\alpha +0.1079L-4.3579$$ (2)

Here, H is the mining depth, (m); *α* stands for the dip angle of the coal seam, (°); L is the length of the working face, (m). However, this formula did not consider floor damage resistance ability, and influence of faults in the working area, etc. Shi[20] proposed an improved linear regression formula and a modified non-linear regression formula respectively, which are:

$$h_{\sigma }=-4.3529+0.0123H+0.1815\alpha +0.8470M+0.1099L-7.6457D+7.2754I$$ (3)

$$h_{\sigma }=-2.0234+1.48\times 10^{-26}H^{9}+0.1913\alpha +1.0637M+0.1016L-5.5536D+7.507I$$ (4)

Where, M is thickness of the seam, (m); D is floor hardness; I represents type of faults and crushed zone the working face passed (1 for yes, 0 for no). The non-linear regression formula has higher accuracy in predicting damaged floor depth.

Floor hardness, D in Eq 4, can be calculated by taking the weighted average hardness factor of each floor stratum as shown in Table 1.

**Table 1:** Floor hardness under coal No.4.

Stratum	Thichness/ (m)	*σ*_*c*_/ (MPa)	D
Medium grain Sandstone	1.2	31.3	0.26
Siltdstone	1.7	83.1	1.00
Mudstone	2.6	9.4	0.05
Coal No.5	3.46	6.6	0.04
Mudstone	1	11.3	0.07
Sandstone	3.2	72	0.81
Mudstone	3.8	11.3	0.07
Limestone	4.6	75.9	0.87
Weighted average floor hardness			0.43

So, the floor damage depth induced by mining activity of panel 24301 is 16.7m, which is larger than 5.5 m—the distance between seam No.3+4 and seam No.5. Therefore, seam No.3+4 and seam No.5 meet the criterion for close seams, and are regarded as close seams.

## 3 Characteristics of stress distribution in floor strata under coal pillars

Mining operations excavate coal and rock and cause the initial stress in the surrounding strata to redistribute to reach a new state of equilibrium, resulting stress concentration in the coal pillars and coal/rock layers below. Changing stress in floor strata under coal pillars affect roadway stability greatly [7].

**Fig 3:**
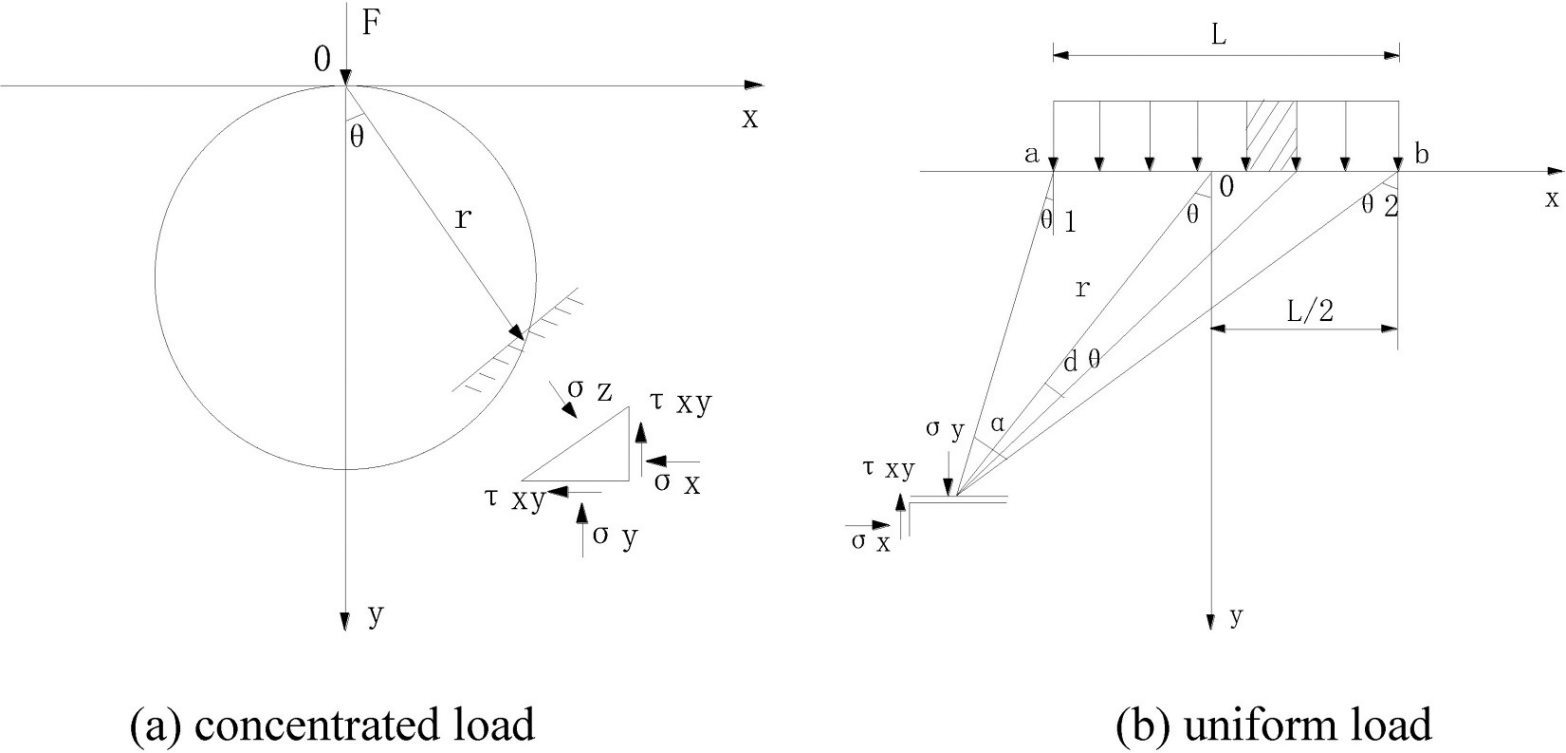
Schematic diagram of simplified mechanical model.

The left coal pillar is subjected to high abutment stress, which is caused by the weight of overburden strata and the weight of partial strata in the gob on one or two sides of the pillar [7]. The stress in the insolated coal pillar’s floor can be regarded as uniform pressure, and is much larger than that of the goaf floor [1]. Moreover, because the width of the coal pillar is much smaller than that of the goaf, the floor can be seen as a semi-infinite elastic rock mass, and the stress of the floor can be simplified as the vertical concentration force in a semi-infinite plane [10]. Thus, the simplified mechanical model of stress under residual pillar can be established, as shown in Fig 3.

Where *L* is the width of the residual coal pillar.

By using Boussinnesq’s solution[21], the stress, induced by concentrated load P of any point (θ, r) in the floor can be expressed in polar coordinates as follows:

$$\sigma_{y}=\frac{2P{cos}^{3}\theta }{\pi r};\;\sigma_{x}=\frac{2P{sin}^{2}cos\theta }{\pi r};\sigma_{z}=\frac{2Psin\theta {cos}^{2}\theta }{\pi r}$$ (5)

In rectangular coordinates:

$$\sigma_{y}=\frac{2P}{\pi }\frac{y^{3}}{{\left(x^{2}+y^{2}\right)}^{2}};\;\sigma_{x}=\frac{2P}{\pi }\frac{yx}{{\left(x^{2}+y^{2}\right)}^{2}};\sigma_{z}=\frac{2P}{\pi }\frac{xy^{2}}{{\left(x^{2}+y^{2}\right)}^{2}}$$ (6)

Where *σ*_*y*_ is the vertical stress; *σ*_*x*_ is the horizontal stress; *τ*_*xy*_ is the shear stress; x represents the distance from the center of the pillar; y represents the depth in the floor.

The stress caused by the uniform stress in the pillar, can be obtained by integrating the stress in Eq (6):

$$\sigma_{y}=\frac{q}{\pi }\left(arctan\frac{b-x}{y}+arctan\frac{b+x}{y}\right)+\frac{2qb(x^{2}-y^{2}-b^{2})y}{\pi (x^{2}+y^{2}-b^{2}+4b^{2}y^{2})}$$ (7)

$$\sigma_{x}=\frac{q}{\pi }\left(arctan\frac{b-x}{y}+arctan\frac{b+x}{y}\right)-\frac{2qb(x^{2}-y^{2}-b^{2})y}{\pi (x^{2}+y^{2}-b^{2}+4b^{2}y^{2})}$$ (8)

$$\tau_{xy}=\frac{4qbxy^{2}}{\pi (x^{2}+y^{2}-b^{2}+4b^{2}y^{2})}$$ (9)

Where q is the uniform stress acting on the floor. Eqs (7), (8) and (9) give analytic expressions for the stress distribution in the floor strata under the isolated pillar. It can be learned from these equations that the impact of the pillar over the lower roadway gradually diminish as the coordinates of the roadway (x, y) grows. In the case of panel 25301, Shaqu No.1 coal mine, the width of the pillar is 50m, and when x is greater than 28.5m, *σ*_*y*_ is less than 0.2q, and the influence of the pillar can be regarded as minor.

## 4 Numerical simulations on roadway layout

There are mainly 3 different layout forms for roadway in the lower coal seam of close seams with upper seam mined: overlapping-type roadway, inboard-type roadway, and outward-type roadway. As shown in Fig 2, the staggered distance, *L*_*1*_, indicates the distance between the centre lines of the upper roadway and roadway of panel 25301. When the roadway is of inboard-type, *L*_*1*_ is a value greater than zero, and *L*_*1*_ = 0 means the roadway is of overlapping-type, while a negative *L*_*1*_ value indicates that the roadways are right under the coal pillar.

### 4.1 Numerical model configuration

Numerical modelling show notable capability to accurately investigate stress distribution under the goaf[22], coal strata deformation[23, 24], and roadway stability[25, 26] in underground excavation. In this paper, we use FLAC3D [27] to study the stress and deformation of roadway of panel 25301 with different staggered distance *L*_*1*_. 11 simulation schemes with staggered distance range from 10m to -10m were developed, as shown in Table 2.

**Table 2:** Numerical simulation scheme of roadway’s layout.

Simulation scheme	1	2	3	4	5	6	7	8	9	10	11
Staggered Distance *L*_*1*_ /(m)	10	8	6	4	2	0	-2	-4	-6	-8	-10

**Fig 4:**
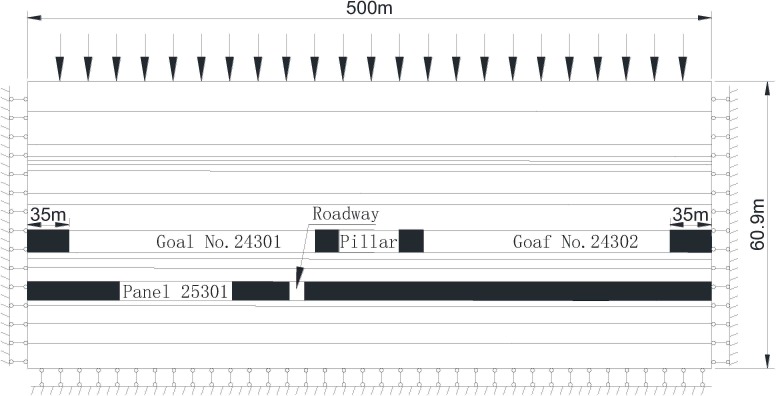
Numerical model.

The simulation models are established by considering the geological and mining conditions of 24301, 24302 and 25301 working face as well as boundary condition of the numerical model. The strike length of the panels (Y-axis) is 60m, and dip length of the model (X-axis), including two panels and a pillar, is 500m, and the height of the model (Z-axis) is 60.9m, as shown in Fig 4. The ideal elastic–plastic constitutive model Mohr Coulomb failure criterion is adopted for the material. Since the coal seams are nearly flat, for the purpose of simplicity, the seams in the model were set to be horizontal. In the numerical model, the horizontal displacement on the border of both sides is fixed, and vertical movement of the bottom boundary is stationary. At the top of the model, an evenly-distributed load of 6.3 MPa is applied to substitute the weight of overlying soil.

The cross section of roadway was 4.0 m×3.46 m, and adopted a uniform upwards pressure of 1MPa on the roof to simulate the effect of a bolt and cable combined support. According to the in-site operation, panel 24301 was mined first in the numerical stimulation, followed by panel 24302, with protective coal pillars on both sides of the model. When the roof of panel 24301 and 24302 cave into mined area, roadways of panel 25301 was excavated afterwards according to different staggered distance for each scheme.

### 4.2 Material properties for coal and surrounding rock mass

Suitable input parameters is the key part of a successful numerical modelling[28]. Mohr-Coulmb yield criterion is adopted in the simulations, and its parameters include: bulk modulus *K*, shear modulus *G*, friction angle *φ*, cohesion *c*, and density *ρ*, of which *K* and *G* can be derived from Young’s modulus *E* and Poisson’s ratio *μ*, as shown in Eqs 10 and 11.

$$k=\frac{E}{3(1-2\mu )}$$ (10)

$$G=\frac{E}{2(1+\mu )}$$ (11)

In order to obtain the mechanical parameters of the coal seam and adjacent rock layers, blocks of immediate roof and were taken from the site. Samples were made and tested for tensile, compression, and shear strength in the laboratory, using ISRM recommended methods[29]. The lithology and mechanical parameters of the model are shown in Table 3.

**Table 3:** Mechanical parameters for rock and coal seams.

Lithology	Cohesion c(MPa)	Frcition angle φ (°)	Bulk module *K* (GPa)	Shear module G (GPa)	Density ρ(g/cm^3^)	Thickness (m)
Coal No.3+4	1.82	40	1.01	0.58	1.36	6.05
Medium grain Sandstone	4.78	37	3.68	1.05	2.62	0.64
Siltstone	5.89	35.1	4.29	2.57	2.71	1.70
Mudstone	1.73	32.2	0.37	0.24	2.13	2.60
Coal No.5	1.84	31.7	0.56	0.26	1.72	3.46
Sandy mudstone	1.30	42	1.60	0.49	2.48	1.00

### 4.3 Numerical simulation results

#### (1) Surrounding rock deformation and layout of the roadways

**Fig 5:**
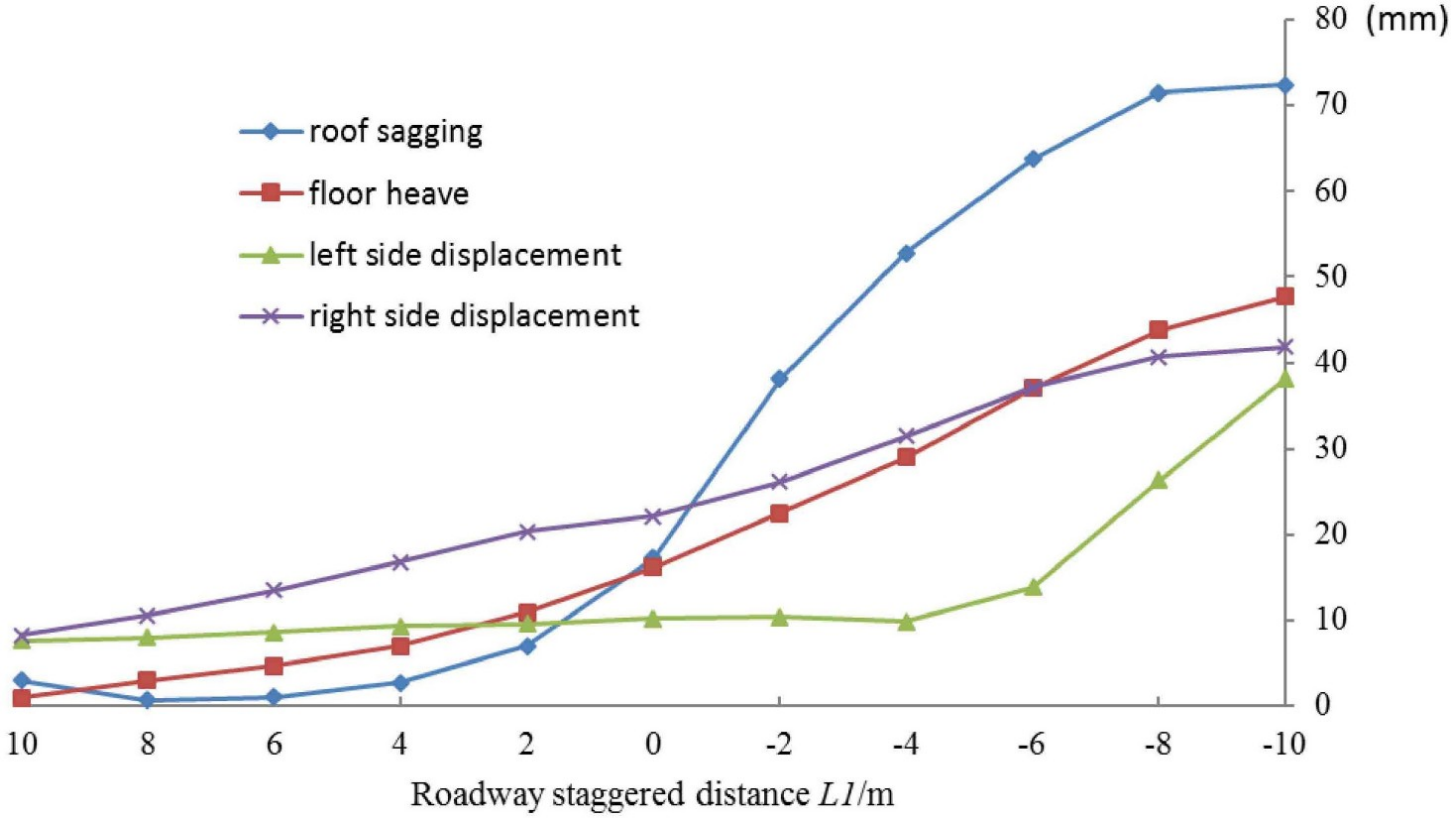
Roadway deformation with different staggered distance.

The displacement of the roof, floor and both sides of the staggered roadway after excavation is shown in Fig 5. Roadways deformation varies with different staggered distance. When the roadway is under the goaf area, that is *L1*>0, the deformation is relatively small. And due to the influence of the left pillar, the right side of the roadway, has greater displacement than that of the left side. In general, the deformation of the roadway increases gradually as the distance between roadway and the center of upper coal pillar decreases, which is in accordance with the stress distribution in the pillar’s floor strata. When the staggered distance *L1* is less than 2m, the roadway’s deformation, especially the roof sagging, increase steeply. The floor heave and left side displacement increase in almost a linear way to around 50mm, as the staggered distance decrease to -10m. When the roadway is under the left pillar, the roof sagging is more than seven times larger than that of under the goaf, thus the roof of the roadway would be hard to support.

#### (2) Evaluation of the plastic failure zone and deformation vector characteristics of schemes

**Fig 6:**
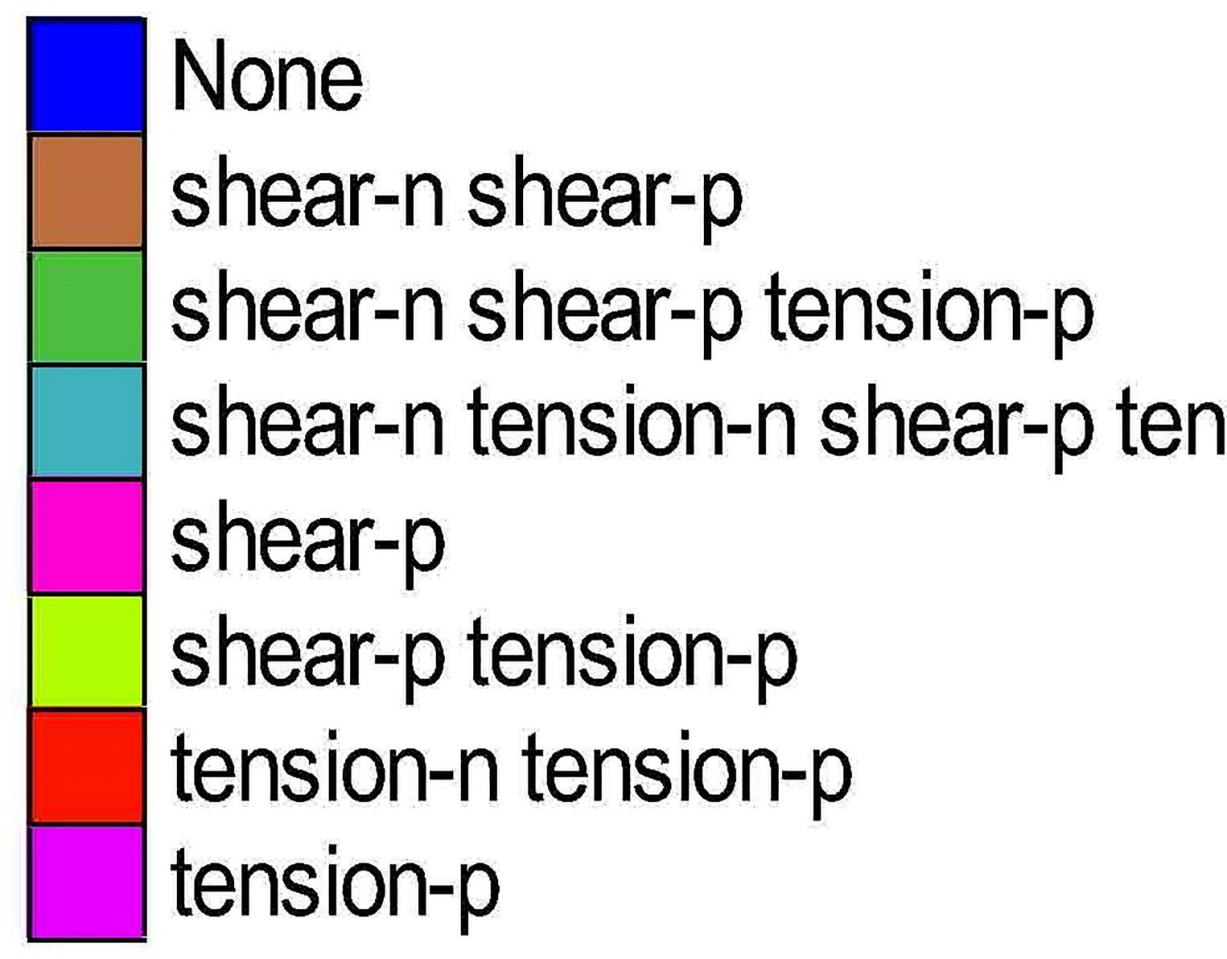
Color for various plastic failure.

**Fig 7:**
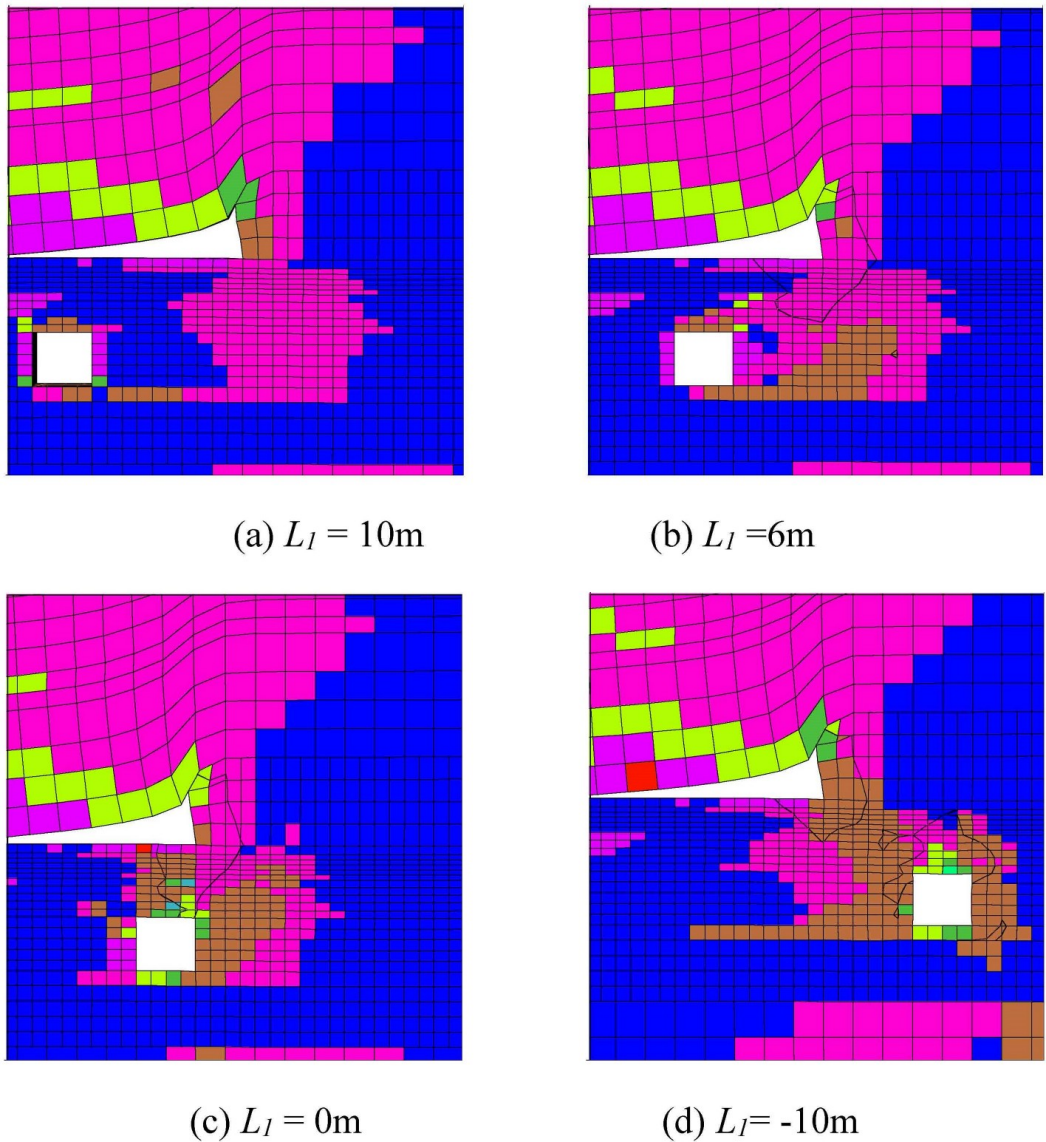
Simulation of plastic zones in different schemes.

**Fig 8:**
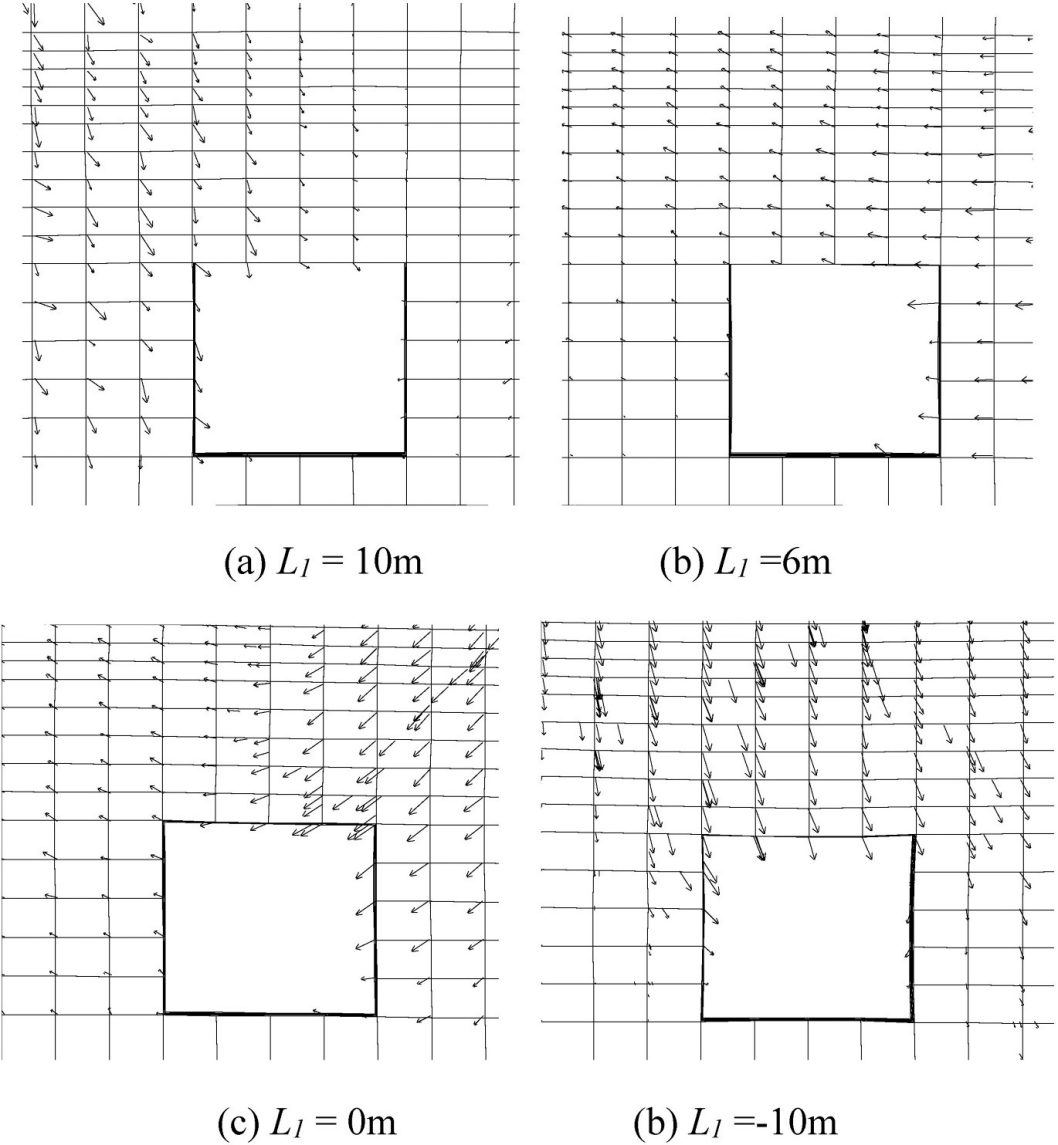
Deformation vectors in plastic zones of schemes.

FLAC3D use ‘n’ to represent plastic failure in current time, and ‘p’ for past time. The specific color for various plastic failure is shown in Fig 6. And the surrounding rock’s plastic failure situation and deformation vector in the failure area is shown in Fig 7 and Fig 8

When the staggered distance *L*_*1*_ decreases from 10m to -10m, the whole plastic zone of the roadway’s surrounding rock gradually increase, when plastic zone under coal pillar remains unchanged. For scheme 1, the extent of roadway’s plastic zone is merely 1m deep into the surrounding rock in roof, floor and both sides, and there is 5m of intact rock between roadway’s plastic zone and plastic zone under coal pillar. When the staggered distance is smaller than 6m, plastic zone in the roadway’s surrounding rock is connected with plastic zone under pillar, causing the plastic zones to merge together. This may lead to rib spalling when the roadway is influenced by abutment pressure. When the roadway is right under the coal pillar, the amount of roof sagging is over 3 times larger than that of when the roadway is right under upper roadway, and this will cause the roadway’s great support difficulties in mining process.

As the roadway come close to the coal pillar, the deformation and plastic zone distribution in the surrounding rock is clearly asymmetrical. The deformation and plastic zone of the roadway’s left side is under better stress and plastic situation compared to the right side. This is because coal pillar in upper seam exert great influence on the deformation and plastic zone distribution for roadway in lower seam. When the roadway is close to the coal pillar, or under the coal pillar, the vector is from coal pillar to the roadway, indicating that roadway’s deformation is greatly influenced by upper coal pillar.

In a word, the roadway should be laid under the goaf, i.e., the stress relief area, not the pillar, to reduce the deformation of the surrounding rock. Considering the stability of the roadway in excavation and mining and coal recovery rate, a staggered distance of 6m is optimal for panel 25301 of Shaqu No.1 coal mine.

## 5 Engineering practice

According to the results of numerical simulation, the optimal layout for roadway of panel 25301 is that the roadway be under the goaf area of panel 24301 with a staggered distance of 6.0m. The Engineering practice was carried out in tailgate of panel 25301 with the optimal roadway layout. And, the length of the mining panel 25301 is 1153.3 m, the workface length is 190 m. And there are no big faults around this panel. The mining operation started in July 2014, with a mining speed of 3.6 m/day.

**Fig 9:**
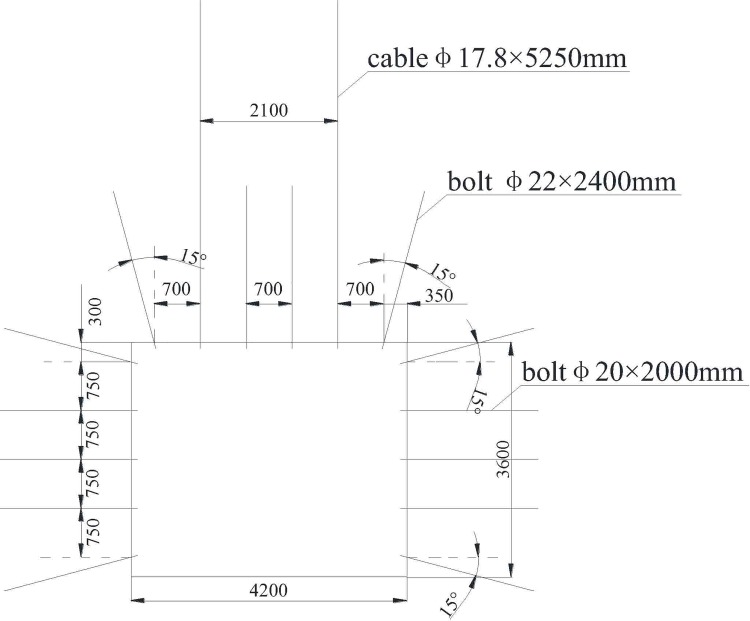
Bolt support system layout.

Fig 9 shows the bolt support system layout of the roadway. Four high strength levorotatory φ22×2400mm bolts were used in the roof and five high strength levorotatory φ20×2000mm bolts in each side. Row×spacing distances of roof and roadway sides were 900×700 mm and 900×800 mm. Cable specification was φ17.8×5250 mm, with a row×spacing distance of 1800×2100 mm.

During the mining operation, in order to evaluate the stability of the roadway, the deformation of the roadway, including displacement of roof and both side walls, were monitored continuously for about four weeks when the face advanced around 80m. Several monitoring sites were set up ahead of the working face with an interval of 50m. Each site recorded subsidence of the roof, and both sides. Due to the similar tendencies of monitoring sites, only the data from one of the sites was presented in this paper.

**Fig 10:**
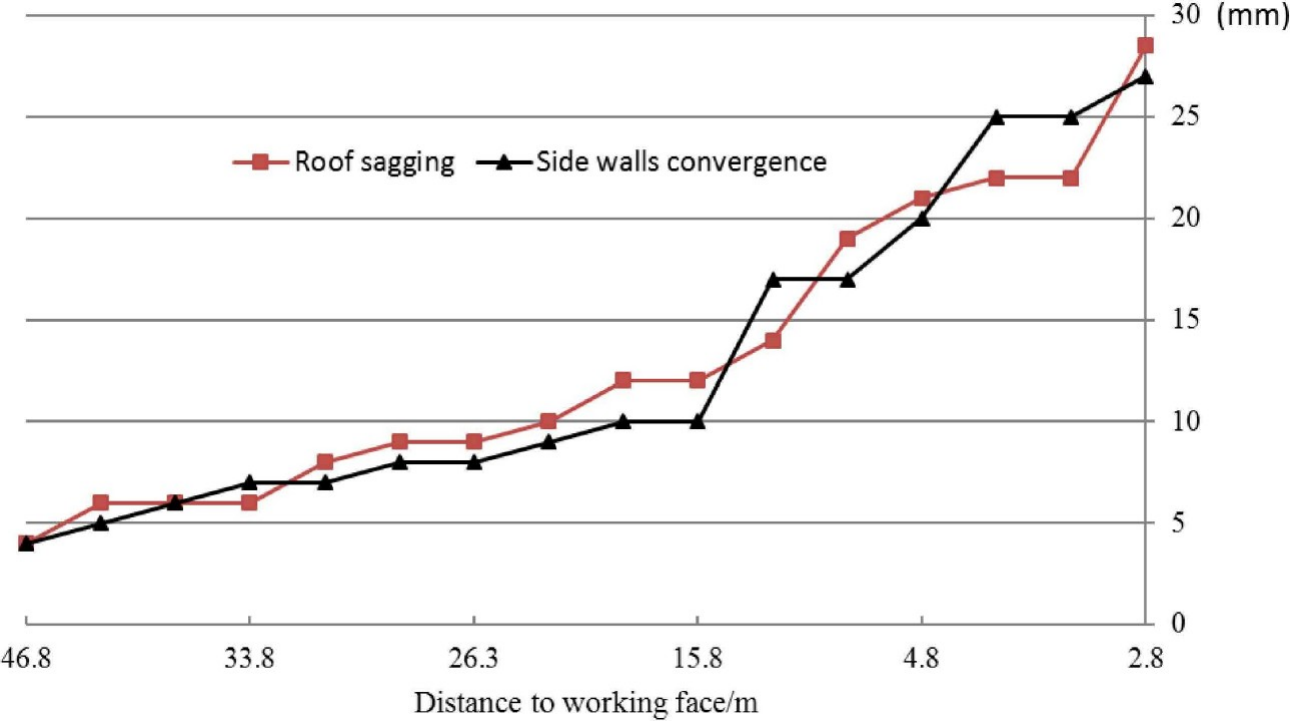
Displacements of roof and side walls.

**Fig 11:**
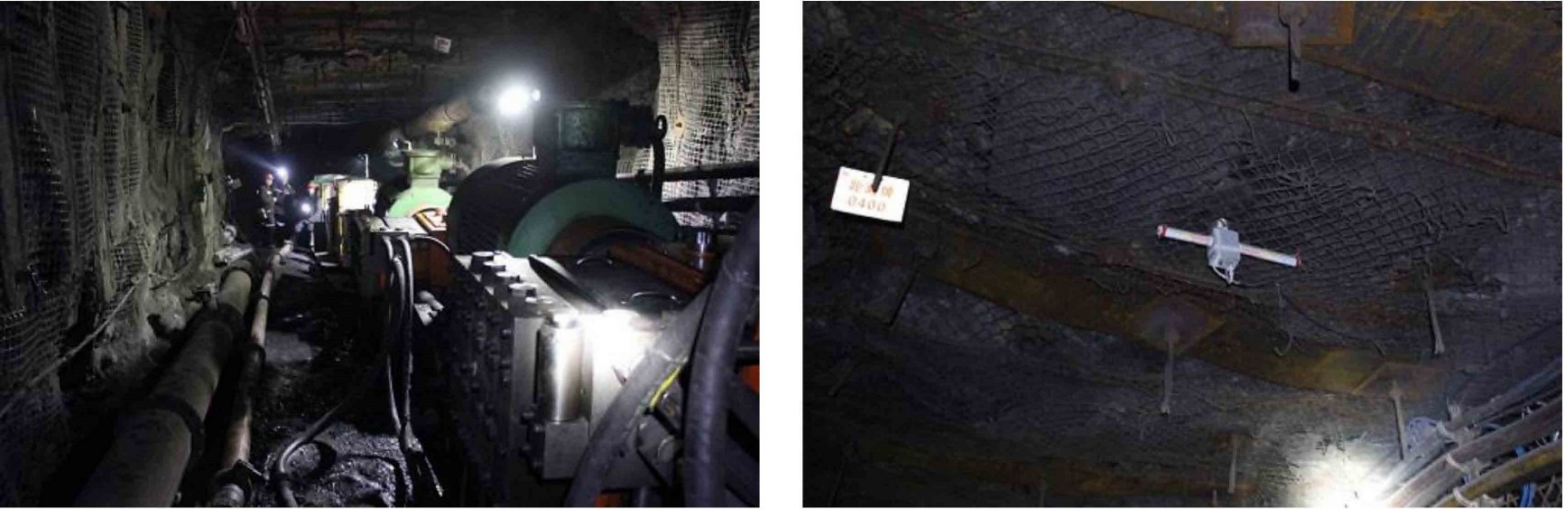
Deformation situation of roadway in panel 25301.

As indicated by Fig 10, the subsidence of the roof and convergence of both sides in the mining period increase gradually in a near-linear way to below 30mm, as the working face advance to the monitoring site. Numerical simulation shows that with a staggered distance of 6m, the roadway’s roof sagging is smaller than monitored value, while side deformation is nearly equivalent to that of the real value. This is partially caused by that in the numerical simulation we use a uniform load on the roof to simulate the effect of bolt support, thus suppress the sagging of the roof. The simulation and shows that because the influence of the coal pillar on the roadway in minor and it is in the stress-relief area, thus the surrounding rock is with little plastic failure, as shown in Fig 11.

Overall, the theoretical analysis and numerical indicate roadway in the stress relief area experience little deformation in excavation process, and can remain stable for the advancing phase of the panel. The engineering practice shows great roadway stability in mining process, with little deformation and plastic failure in the surrounding rock mass, thus validates the numerical simulations conducted earlier.

## 6 Conclusions

1. In the mining of close coal seams, especially for ultra-close seams, coal pillar left in upper seam not only cause stress concentration in the floor strata including lower coal seams, but left massive coal unmined in the goaf as well. Thus, the most resources-exploitation-efficient method is with narrow or none at all coal pillars.
2. The deformation of bare roadway with different layout schemes was comparatively analysed, through numerical simulations. The results show that the roadway of interest is under better condition in aspect of deformation and stress when the roadway is in-board. And considering the roadway stability and coal recovery rate, the optimal layout of the roadway, is with a staggered distance of 6m.
3. Under the current support system and the optimized roadway layout, the roadway’s deformation is blow 30mm during the whole process of mining.

## Supporting information

S1 File. Mine pressure observation data of panel 25301. (XLSX)

S2 File General stratigraphic log of panel 25301. (DWG)

S3 File Roadway layout of panel 25301. (DWG)
